# Integrating QTL mapping, BSA-seq and RNA-seq to identify candidate genes regulating seed storability from Dongxiang wild rice

**DOI:** 10.3389/fpls.2025.1644153

**Published:** 2025-08-13

**Authors:** Shiqi Zhou, Ting Wu, Shilin Wang, Jiwai He, Biaolin Hu

**Affiliations:** ^1^ Rice Research Institute, Jiangxi Academy of Agricultural Sciences, Nanchang, China; ^2^ College of Agronomy, Hunan Agricultural University, Changsha, China

**Keywords:** Dongxiang wild rice, seed storability, RNA-seq, BSA-seq, haplotype

## Abstract

Rice seed storability (SS) is crucial for germplasm preservation, agricultural production, grain storage, and food security. Dongxiang wild rice (*Oryza rufipogon* Griff., hereafter DXWR) is a common wild rice adapted to the northernmost area worldwide and possesses strong SS. Xieqingzao B (XB) is a maintainer line widely used in hybrid rice. DXWR and XB were crossed and subsequently backcrossed with XB four times to develop the strong -SS line 19H19 in the BC_4_F_2_. Subsequently, 19H19, XB, and their 120 BC_5_F_2_ lines were employed to study SS under artificial aging. A genetic map identified four quantitative trait loci (QTLs), and BSA-seq identified another four QTLs; *qSS6.1* was co-identified by both approaches. By combining QTL mapping, BSA-seq, and RNA-seq, 31 candidate genes were identified in total for SS. Among these, the gene *Os06g0287500* within the *qSS6.1* interval was associated with “defense response.” It was downregulated in 19H19 but upregulated in XB after aging, likely due to nonsynonymous mutations and deletions in the exon of parental XB. Genetic analysis confirmed that *Os06g0287500* was significantly associated with SS in rice. Haplotype analysis of *Os06g0287500* among 141 core germplasm accessions revealed that the Hap1/DXWR(19H19)-type accessions had significantly stronger SS than the Hap2/XB -type and Hap3 accessions under artificial aging. The Hap1/DXWR (19H19) group exhibited the strongest SS among the three haplotypes under both artificial and natural aging conditions. A gene interaction network regulating SS in rice was constructed based on a STRING database assay, wherein differentially expressed genes (DEGs) related to “kinase activity” interacted with *Os06g0287500*. Therefore, *Os06g0287500* is a promising candidate gene involved in SS in rice. These findings not only provide critical insight into the genetic mechanisms regulating SS in rice but also offer novel genetic resources for broadening the gene pool of cultivated rice and developing new varieties with enhanced SS through QTL pyramiding.

## 1 Introduction

Rice (*Oryza sativa* L.) is one of the most important food crops, feeding more than half of the global population (Zhou et al., 2024a). Therefore, stable production associated with a sustainable supply of rice plays a pivotal role in global food security. However, seed deterioration during storage is a natural and irreversible process that leads to a drastic decline in both edible quality and seed vigor, posing a serious challenge to grain production. In China, postharvest seed deterioration leads to an annual loss of 6.7 million tons of rice, creating significant challenges for food security (Zhou et al., 2024a). Although modern technology can effectively control temperature, humidity, and atmosphere in warehouses to protect grain quality and prolong seed longevity (Ziegler et al., 2021), such protection incurs substantial energy costs that are unaffordable in most developing countries. Comparatively, genetic improvement of seed storability (SS) through breeding approaches can help conserve energy while maintaining seed viability during storage, which is particularly beneficial in mitigating the impact of climate change.

Naturally, SS varies greatly among different rice germplasm accessions (Lee et al., 2019), and such variation provides an essential foundation for improving SS through genetic approaches. Rice SS, essential for grain viability, storability, and quality, is controlled by multiple genes (Wang et al., 2022a). Therefore, developing cultivars from germplasm with strong SS is a feasible strategy with great significance for ensuring food quantity and quality after harvest.

Based on seed germination percentage under either natural or artificial aging, more than 120 quantitative trait loci (QTLs) for SS have been identified to date using various biparental populations in rice. These QTLs are distributed across all 12 chromosomes, with the majority located on chromosome 9 (Zhou et al., 2024a; Dong et al., 2017). Among them, more than 95 QTLs with favorable *indica* alleles have been used to effectively improve SS. These were identified from various mapping populations involving *indica–japonica* crosses, indicating stronger SS in the *indica* subspecies compared with *japonica* (Wu et al., 2021).

The major QTL *qLG-9* was first detected for SS (Miura et al., 2002; Li et al., 2012), followed by several others, including *RC9-2*, *qGP-9*, *qRGR-9*, *qMT-SGC9.1*, *qSS-9*, *qSSn-9*, and *qDT-SGC9.1* (Li et al., 2012, Li et al., 2017; Lin et al., 2015; Sasaki et al., 2005, Sasaki et al., 2015; Xue et al., 2008; Jiang et al., 2011), all of which overlap with *qLG-9*, indicating that this QTL hotspot is important and reliable for SS in rice.

Among these, only a few major QTLs have been subsequently fine-mapped, such as *qGP-9/qLG-9*/*qSS-9* (Li et al., 2012, Li et al., 2017; Lin et al., 2015; Yuan et al., 2019), *qSS1*, and *qSS3.1* (Yuan et al., 2019, Yuan et al., 2021). To date, four have been cloned and functionally characterized: the fatty acid hydroxylase gene *OsFAH2* in *qSS3.1* (Yuan et al., 2019); the indole-3-acetic acid (IAA) amine synthase gene *OsGH3–2* in *qSS1* (Yuan et al., 2021); the *copper/zinc superoxide dismutase 2* (*OsCSD2*) in *qSL3*; and the candidate gene *OsCSD3* in *qSL7.2* (Zheng et al., 2024b).

For instance, overexpression of *OsFAH2* enhances seed storability, while *OsGH3–2* catalyzes the conjugation of IAA and amino acids to form inactive auxin, acting as a negative regulator of SS (Yuan et al., 2019, Yuan et al., 2021). *OsCSD2* and *OsCSD3* are copper/zinc superoxide dismutases that regulate SS by modulating antioxidant enzymes and abscisic acid (ABA); their overexpression enhances SS (Zheng et al., 2024b). Additionally, the *Rc* gene plays a key role in improving tolerance to partial pressure of oxygen aging in dry grains (Prasad et al., 2023).

Functional analysis of these SS-related genes has provided valuable insight into the physiological, biochemical, and molecular mechanisms of SS in rice. However, most SS QTLs and genes have been identified from biparental mapping populations derived from crosses among cultivars or landraces, and only a few originate from interspecific crosses between wild and cultivated rice (Jin et al., 2018; Zhao et al., 2021). Numerous studies have concluded that modern rice varieties generally have lower SS than landraces and wild rice, especially hybrid rice (Zhao et al., 2021; Chen et al., 2022a; Zhou et al., 2023). This may be due to the loss of beneficial genes or alleles during artificial selection, domestication, and long-term evolution (Xu and Sun, 2021; Wang et al., 2022c). Therefore, it is essential to further explore and utilize the natural variation in SS from wild progenitors to enhance our understanding of the genetic and molecular mechanisms required for breeding hybrid rice with strong SS.

Wild rice progenitors serve as a vital gene reservoir for expanding the beneficial genetic base of modern cultivars due to their long-term adaptation to diverse biogeographic environments and evolution of resistance to various biotic and abiotic stresses (Xu and Sun, 2021). Dongxiang common wild rice (*Oryza rufipogon* Griff., hereafter DXWR) is such a progenitor, harboring abundant genes associated with multiple stress tolerances (Zhao et al., 2021; Hu et al., 2016), including extremely strong SS (Zhao et al., 2021; Zhou et al., 2023; Jiang et al., 2010). This makes DXWR a unique genetic pool for mining and exploiting valuable SS-related genes to improve rice cultivars. However, the molecular mechanism underlying SS in DXWR remains unclear, which limits its effective use in breeding. To address this issue, we performed an interspecific cross between Xieqingzao B (XB) and DXWR. The progeny were backcrossed with XB, and a selected BC_1_ F_10_ line was continuously backcrossed to yield 19H19 in the BC_4_F_2_ generation. In the present study, we used 19H19 and its BC_5_F_2_ segregation population with XB for genotyping, employing a high-density bin map in combination with BSA-seq to detect QTLs related to SS. Additionally, transcriptome profiling of 19H19 and its recurrent parent XB before and after artificial aging was conducted to identify differentially expressed genes (DEGs) and candidate genes associated with SS. Our objective was to elucidate the genetic mechanism of SS in DXWR and identify favorable *O. rufipogon* alleles for improving SS through QTL pyramiding.

## 2 Materials and methods

### 2.1 Material preparation and phenotyping

The rice material used was a cross between XB and DXWR, followed by backcrossing and selfing to screen out the line 19H19, which was identified to have the strongest SS after three rounds of evaluations under artificial aging with 42°C and 80% relative humidity for 28 days. Subsequently, 19H19 was backcrossed with XB and self-pollinated to obtain the 120 BC_5_F_2_ lines (**Supplementary Figure S1**) (Hu et al., 2016; Zhou et al., 2024b).

Fifty seeds were sampled from 19H19, XB, and each of their 120 BC_5_F_2_ lines for artificial aging at 42°C and 80% relative humidity for 28 days, with moisture controlled by an LH-150 (Jiangsu, China) thermostatic moisture regulator. The treated samples were then placed under ideal conditions at 30°C and 80% relative humidity for germination rate scoring. The experiments were replicated three times. Statistical analysis of germination rate was conducted using the method proposed by Zhou et al. (2022).

### 2.2 Bioinformatical procedures analysis

Leaf samples were taken from 19H19, XB, and each of the 120 BC_5_F_2_ lines at the seedling stage. Genomic DNA was extracted from three bulked leaves per line using the CTAB method (Chaparro-Encinas et al., 2020). The DNA library was constructed and sequenced using the genotyping -by -sequencing (GBS) protocol with PE125 reads on the NovaSeq6000 platform (Illumina, San Diego, CA). Clean reads were obtained by filtering raw reads according to the rules set by Guo et al. (2022). The detection of single-nucleotide polymorphisms (SNPs) was conducted using the method described by Li and Durbin (2009). QTL detection was performed using R/QTL software version 1.39-5 (Arends et al., 2010). A logarithm of odds (LOD) threshold of 2.5 or higher was used to declare a putative QTL in each bin, based on a 0.05 significance probability. The regional genes were annotated and analyzed using the method developed by Zhou et al. (2024b).

### 2.3 BSA-seq analysis

Four DNA libraries were constructed from total DNA samples of S-bulks (20 individuals with strong SS, germination rate 80–100%), W-bulks (20 individuals with weak SS, germination rate 0–15%), and their parents 19H19 and XB. The sequencing library was generated using the TruSeq Nano DNA HT Sample Prep Kit (Illumina, USA) following the manufacturer’s recommendations, and the four samples were labeled as S, W, 19H19, and XB. Raw reads with low quality (mean Phred score< 20), adapter contamination, or unrecognizable nucleotides (N base >10%) were trimmed or discarded using the software Fastp (Chen et al., 2018). The resulting clean reads were mapped to the reference genome using BWA-MEME software (version Bwa-mem 2-2.1) under default mapping parameters (Jung and Han, 2022; http://plants.ensembl.org/Oryza_sativa). Germline variant calling, including SNPs and InDels across all samples, was performed using the Haplotyper and GVCFTyper programs in Sentieon Genomics Tools (Freed et al., 2017). The SNPs and InDels were categorized based on their chromosomal positions (e.g., exons and 1-kb upstream regions) and effects (e.g., missense, start codon gain or loss, stop codon gain or loss, and splicing mutations). The Euclidean distance (ED) algorithm was employed to perform BSA analysis (Hill et al., 2013). Only windows with an average ED above the 99.5% confidence interval threshold were considered as candidate regions. To demonstrate nucleotide sequence differences in the identified candidate regions between XB and 19H19, the regions were sequenced using the method described by Zhou et al. (2024b). Validation of InDel markers for SS was performed using the default settings of the BIP (QTL mapping in biparental populations) approach in IciMapping version 4.2 (Meng et al., 2015).

### 2.4 RNA sequencing and RT-qPCR analysis

RNA was extracted from tissue samples of XB and 19H19 before and after aging treatment using the R6827 Plant RNA Kit. The concentration, purity, and integrity of the extracted RNA were assessed using a NanoDrop One spectrophotometer (NanoDrop Technologies, Wilmington, DE). After the estimation, oligo (dT)-attached magnetic beads were used to enrich mRNA from total RNA, followed by fragmentation. Then, the first- and second-strand cDNA synthesis was performed by reverse transcription, followed by end repair, addition of A tails, attachment of sequencing adapters, purification, and PCR amplification to complete the entire library preparation. Libraries that passed quality checking were pooled to the flow cell according to the required effective concentration and target data volume. Sequencing was performed on the Illumina NovaSeq high-throughput platform after clustering with cBOT. Low-quality reads were removed, and clean reads were retained for subsequent analysis (Chen et al., 2018). Quality control of the clean reads was conducted using FastQC (version: 0.11.9; default parameters) (Andrews, 2012). Transcriptome sequences were aligned to the reference genome following the protocol of Shang et al. (2023). Statistical analysis was conducted using STAR (version: 2.7.9a; default parameters), and gene expression significance was evaluated using p-values (Dobin et al., 2013). Genes with *p*< 0.01 and an absolute foldchange ≥ 2 were considered significantly differentially expressed.

In parallel, the same total RNA samples were reverse-transcribed using the FastKing RT Kit (Tiangen). RT-qPCR was then performed using the ChamQ Universal SYBR qPCR Master Mix (Vazyme), with *OsACTIN* as the internal reference. Primers used for RT-qPCR are listed in **Supplementary Table S1**. All RT-qPCR experiments were conducted with three biological replicates.

### 2.5 Network analysis

A gene–gene interaction network was constructed to predict the interactions between those genes of interest identified by QTL mapping, BSA-seq, and the DEGs using the STRING (version 11.0), a gene interaction prediction database.

### 2.6 Haplotype analysis of candidate genes

A total of 141 and 127 core accessions from a collection of 3,000 rice (*Oryza sativa*) accessions constructed by the Institute of Crop Sciences, Chinese Academy of Agricultural Sciences (3K) (Wang et al., 2018), were subjected to artificially aging and naturally aging treatments (He et al., 2022), respectively. The natural and artificial aging conditions were a room at about 30°C and 60%–80% relative humidity for 18 months and at 45°C and 95% relative humidity for 7 days. The complete coding sequences of the 19H19 and XB candidate genes were sequenced, and all non-synonymous mutation SNPs and InDels were extracted. Haplotype analysis was conducted on those accessions with variations consistent with 19H19 or XB, using the online RiceVarMap v2.0 database (http://ricevarmap.ncpgr.cn).

## 3 Results

### 3.1 Phenotypic SS in BC_5_F_2_ BIL population and their two parents

**Figure 1:**
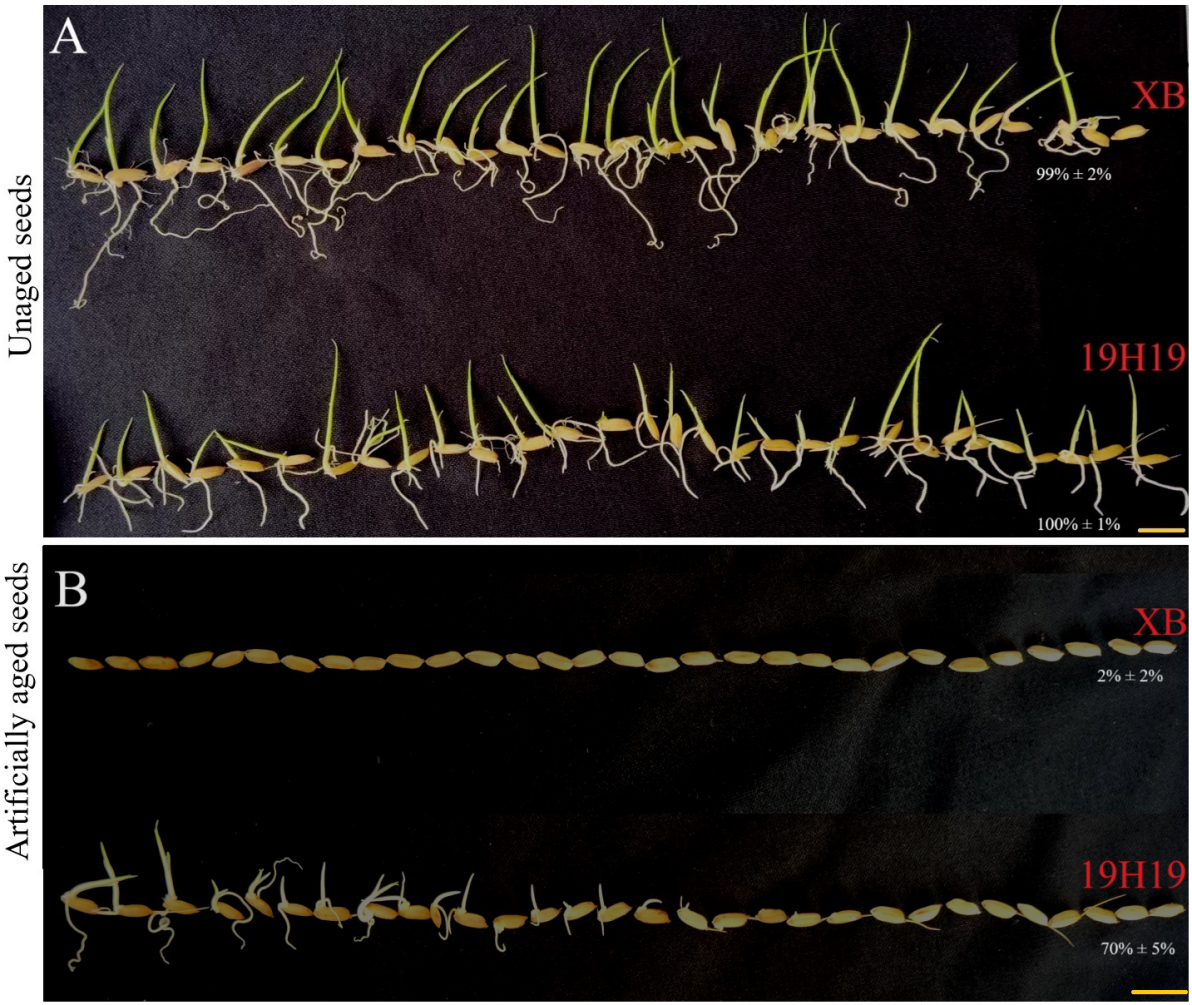
Phenotypes of unaged and artificially aged seeds of 19H19 and XB. Unaged seeds of 19H19 and XB are shown in panel **(A)**, and seeds aged under artificial aging conditions (42°C and 80% relative humidity for 28 days) are shown in panel **(B)**. Scale bars = 1 cm.

Genetically, 19H19 exhibited a high genetic homogeneity of 82.9% with XB at 3,450,753 SNPs in the XB background since it was a BIL derivative from XB (Zhou et al., 2024b), but it showed stronger SS than XB. Under the normal conditions, both 19H19 and XB had a similarly high germination rate (GR), with 100% and 99%, respectively (**Figure 1A**). However, after artificial aging at 42°C and 80% relative humidity for 28 days, 19H19 still maintained a high GR of 70%, while XB almost lost its capability of germination with a GR of 2% (**Figure 1B**). Obviously, the artificial aging made XB almost completely lose its seed vigor, or SS, while 19H19 still maintained its very high seed vigor, or SS.

**Figure 2:**
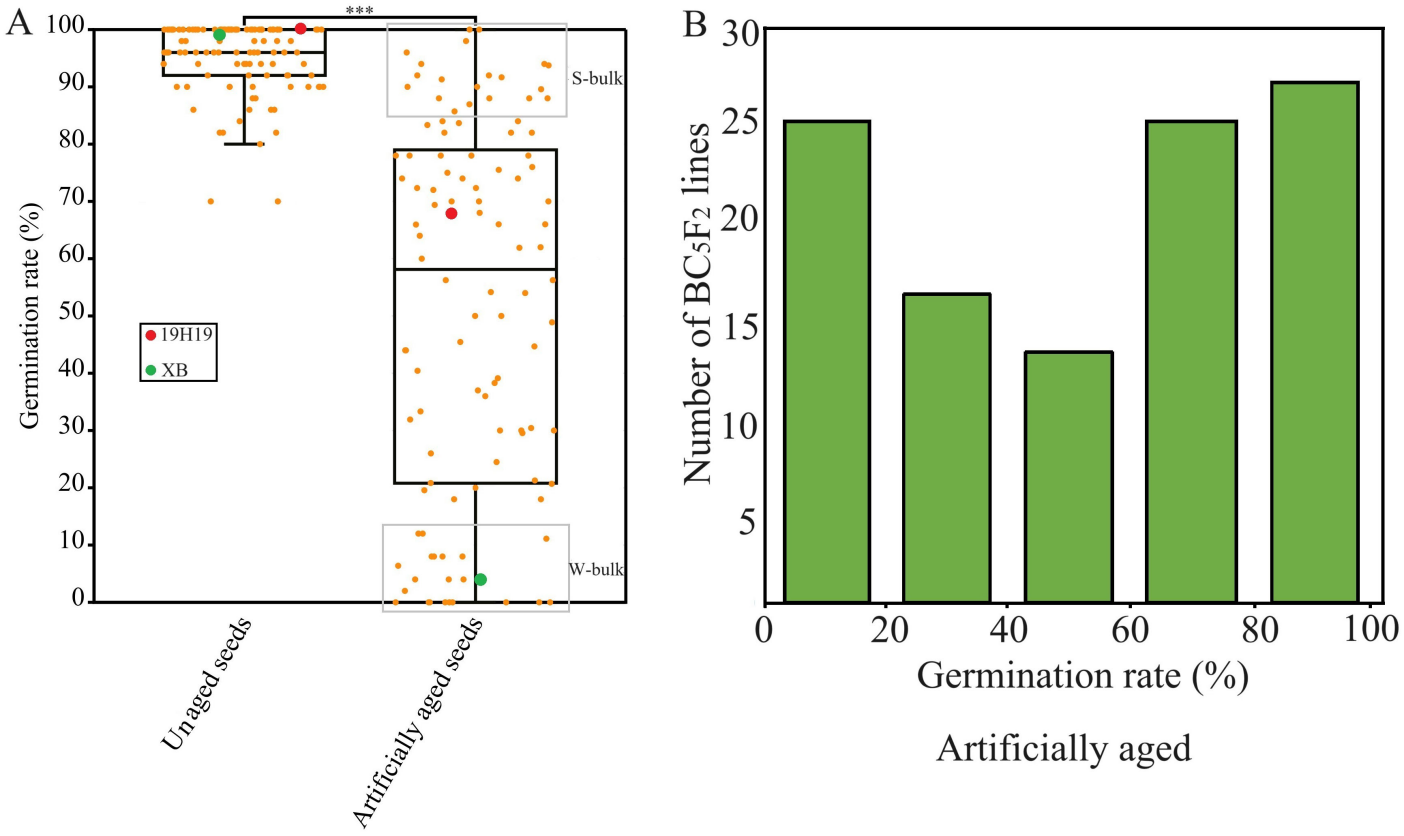
Seed germination of the BC_5_F_2_ backcross inbred line (BIL) population and their parental lines under artificial aging for 28 days. **(A)** Boxplot showing the distribution of germination rates (GRs) among BILs and parental lines after artifical aging, ***P<0.001. **(B)** Frequency distribution of GR values representing seed storability under the same aging conditions.

Under normal conditions, the GR values ranged from 70% to 100% in the BIL population, with an average of 95.5%, indicating that each of the 120 BILs had a normally high seed vigor originally (**Figure 2A**). However, after the aging, the GR values ranged from 0% to 100% in the BIL population, with an average of 58.5%. A continuous distribution and transgressive segregation of GR in the population exhibited a typical pattern of quantitative inheritance (**Figure 2B**).

From the population, we sampled 20 BILs with GR greater than 80% to build an S-bulk and another 20 BILs with GR lower than 15% to build a W-bulk from each of three artificial aging treatments, respectively, for BSA-seq (**Figure 2A**).

### 3.2 QTL mapping identified *qSS6.1* for SS in the BIL population

**Figure 3:**
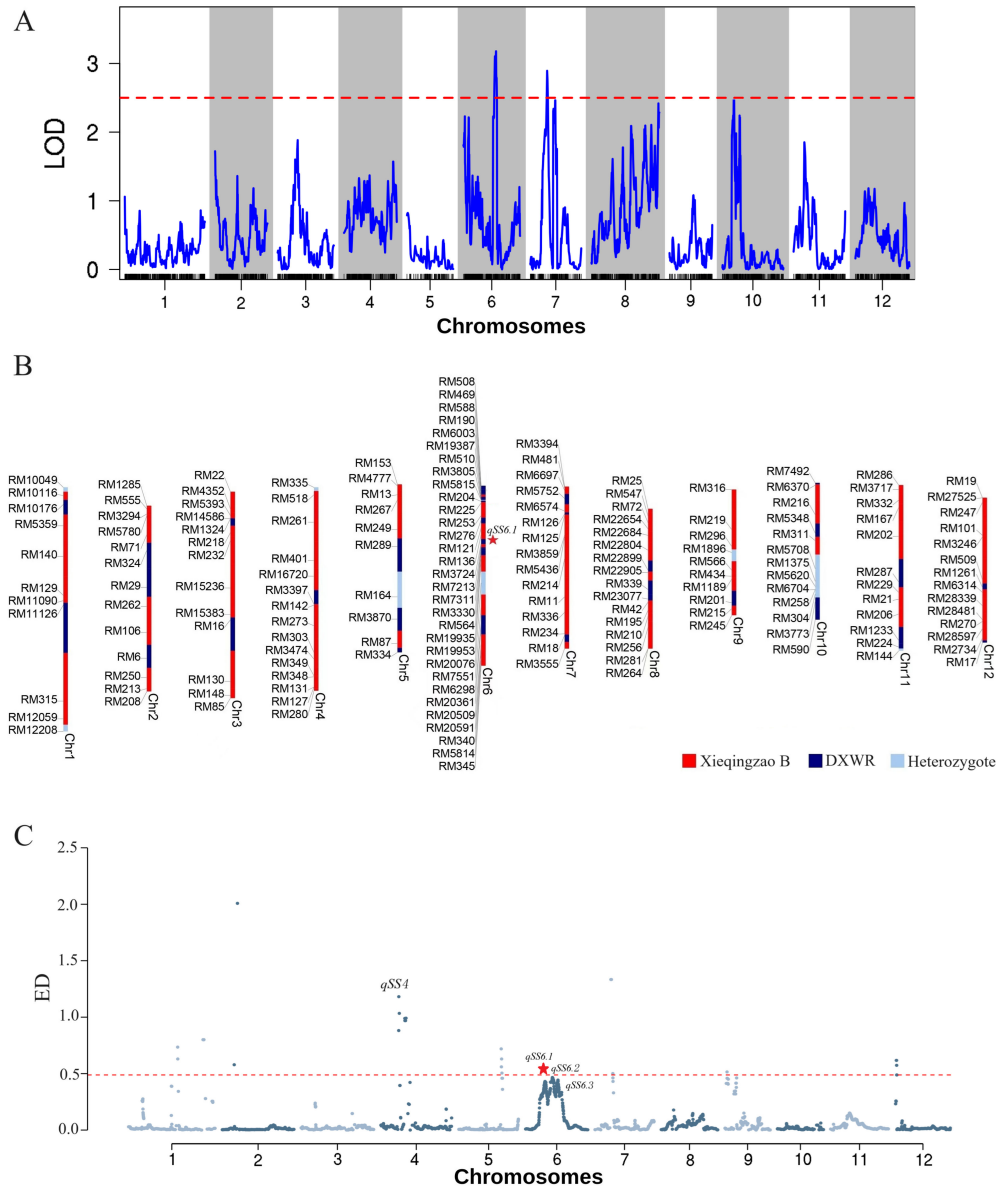
QTL mapping and BSA-seq results associated with seed storability after artificial aging. **(A)** QTL mapping analysis of germination rate (GR) using a high-density SNP bin map. **(C)** Genomic regions identified by BSA-seq. **(B)** Genotype of 19H19 indicating loci contributing to enhanced seed storability.

A genetic map composed of 2,059 SNP bin markers was built to perform QTL mapping for SS measured by GR under artificial aging. This linkage map spanned 1,645.884 cM across all 12 chromosomes, with an average distance of 0.799 cM between adjacent markers (**Supplementary Figure S2**; **Supplementary Table S2**). Using a threshold of logarithm of the odds (LOD) ≥ 2.5, four QTLs for SS on chromosomes 6, 7, and 10, respectively, were declared at a significance level of 0.05 probability (**Figure 3A**).

Among them, *qSS6.1* had the highest LOD score of 3.2 and explained the greatest phenotypic variation (PVE) at 21.84%, where the DXWR allele increased GR by 12.62% under artificial aging (**Figures 3A, B**; **Table 1**). Both *qSS7.1* and *qSS7.2* on chromosome 7 had LOD scores of 2.9 and 2.5, explaining 14.81% and 4.92% of phenotypic variation, respectively, where the XB allele increased GR by 11.00% and 12.92% under artificial aging. With an LOD of 2.5, *qSS10* accounted for 8.22% of phenotypic variation, where the DXWR allele increased GR by 1.15% under artificial aging.

**Table 1:** QTL summary under aging stress for GR.

QTL	Region	Length. kb	LOD	Add	Dom	PVE
*qSS6.1*	7652428-11096971	3444.543	3.2	12.62	7.88	21.84
*qSS7.1*	2502750-4646359	2143.609	2. 9	-11.00	-2.88	14.81
*qSS7.2*	6062071-7070646	1008.575	2.5	-12.92	3.31	4.92
*qSS10*	3992799-8396755	4403.956	2.5	1.15	18.32	8.22

LOD score, logarithm of odds; PVE (%), percentage of total phenotypic variance explained by individual QTL; Add, additive effect of QTL from DXWR.

### 3.3 BSA-seq analysis confirmed *qSS6.1* for SS

For the four libraries built from RNA extracted from XB, 19H19, W-bulks, and S-bulks, approximately 85,309.37 Mb of raw read data were acquired by BSA-seq, from which 82,676.19 Mbp clean reads (96.91%) were obtained after filtering via the Illumina TruSeq platform. These clean reads were mapped to the reference genome, with effective mapping percentages of 96.59%, 97.02%, 96.74%, and 97.33% for XB, W-bulk, 19H19, and S-bulk libraries, respectively. The sequencing depths were 47.71-, 54.1-, 58.31-, and 54.46-fold for XB, W-bulks, 19H19, and S-bulks, respectively (**Supplementary Table S3**), confirming the accuracy of the BSA-seq analysis.

Using the 99.5% confidence threshold, *qSS4*, *qSS6.1*, *qSS6.2*, and *qSS6.3* were identified as associated with SS. Except for *qSS4* on chromosome 4, the remaining three were located on chromosome 6. *qSS4* spanned 0.52 Mb (11.76–12.28 Mb). *qSS6.1*, *qSS6.2*, and *qSS6.3* spanned 0.42 Mb (9.54–9.96 Mb), 1.47 Mb (12.17–13.64 Mb), and 0.26 Mb (15.52–15.78 Mb), respectively (**Figures 3C**; **Supplementary Table S4**). Notably, *qSS6.1* was located in the same genomic region identified by QTL mapping (**Table 1**; **Supplementary Table S3**), indicating that *qSS6.1* is a relatively stable genomic region for SS in rice, where the favorable DXWR allele significantly increased germination under aging stress (**Table 1**).

A large number of SNPs and InDels were identified in these QTL intervals (**Supplementary Table S5**). We regarded SNPs that caused non -synonymous substitutions or InDels in coding regions, as well as Indels that induced frameshift mutations in gene promoter regions, as effective SNPs or InDels due to their potential phenotypic effects. Within the *qSS6.1* and *qSS6.2* intervals, we identified 54 and 23 effective SNPs and 4 and 3 effective InDels, respectively. Based on these, 25 candidate genes were found in both the *qSS6.1* and *qSS6.2* regions, respectively (**Supplementary Table S4**).

### 3.4 Transcriptomic comparison between 19H19 and XB for SS

The quality data of transcriptomes from 19H19 and XB before and after artificial aging are presented in **Supplementary Table S6**. Transcriptomic Pearson correlation coefficients among the three replicates were all greater than 0.9, confirming both the reproducibility and reliability of the results (**Supplementary Figure S3**).

Comparing the normal and artificially aged conditions among 61,671 genes, 357 and 284 upregulated genes were identified from 19H19 (19H19A vs. 19H19U) and XB (XBA vs. XBU), respectively, of which 61 were common to both. Additionally, 1,481 and 1,261 downregulated genes were identified from 19H19A vs. 19H19U and XBA vs. XBU, respectively, with 351 shared between them (**Supplementary Figure S4**). Among the common genes, seven were upregulated in 19H19A vs. 19H19U but downregulated in XBA vs. XBU, whereas 22 were downregulated in 19H19A vs. 19H19U but upregulated in XBA vs. XBU (**Supplementary Figure S4**).

Gene Ontology (GO) term enrichment and Kyoto Encyclopedia of Genes and Genomes (KEGG) pathway analyses of the commonly upregulated or downregulated genes in 19H19A vs. 19H19U and XBA vs. XBU revealed enrichment in 25 GO terms and 3 KEGG pathways (**Supplementary Figures S5A, B**). These DEGs were induced by the artificial aging treatment. DEGs showing opposite expression trends between 19H19A vs. 19H19U and XBA vs. XBU were selected to explore the molecular mechanisms of aging and were mainly enriched in 15 GO terms and 1 KEGG pathway (**Supplementary Figures S5C, D**). Among them, the ‘endopeptidase inhibitor activity’ and ‘glutathione metabolism’ pathways were associated with SS (Huang et al., 2020; Xia et al., 2020). In addition, enrichment analysis of genes uniquely upregulated or downregulated in 19H19A vs. 19H19U and XBA vs. XBU showed that uniquely downregulated genes in 19H19 and uniquely upregulated genes in XB were enriched in the ‘DNA binding’ and ‘lipid transport’ pathways (**Supplementary Figure S5E**). These unique genes were enriched in 33 GO terms and 14 KEGG pathways (**Supplementary Figures S5E, F**).

### 3.5 Expression profiles of candidate genes for SS

To identify differentially expressed candidate genes associated with SS, we performed gene expression profiling via RNA-seq on the *qSS6.1* interval, which was co-identified by BSA-seq and QTL mapping. As a result, 31 genes showed different expression levels between XB and 19H19 before and after aging.

**Figure 4:**
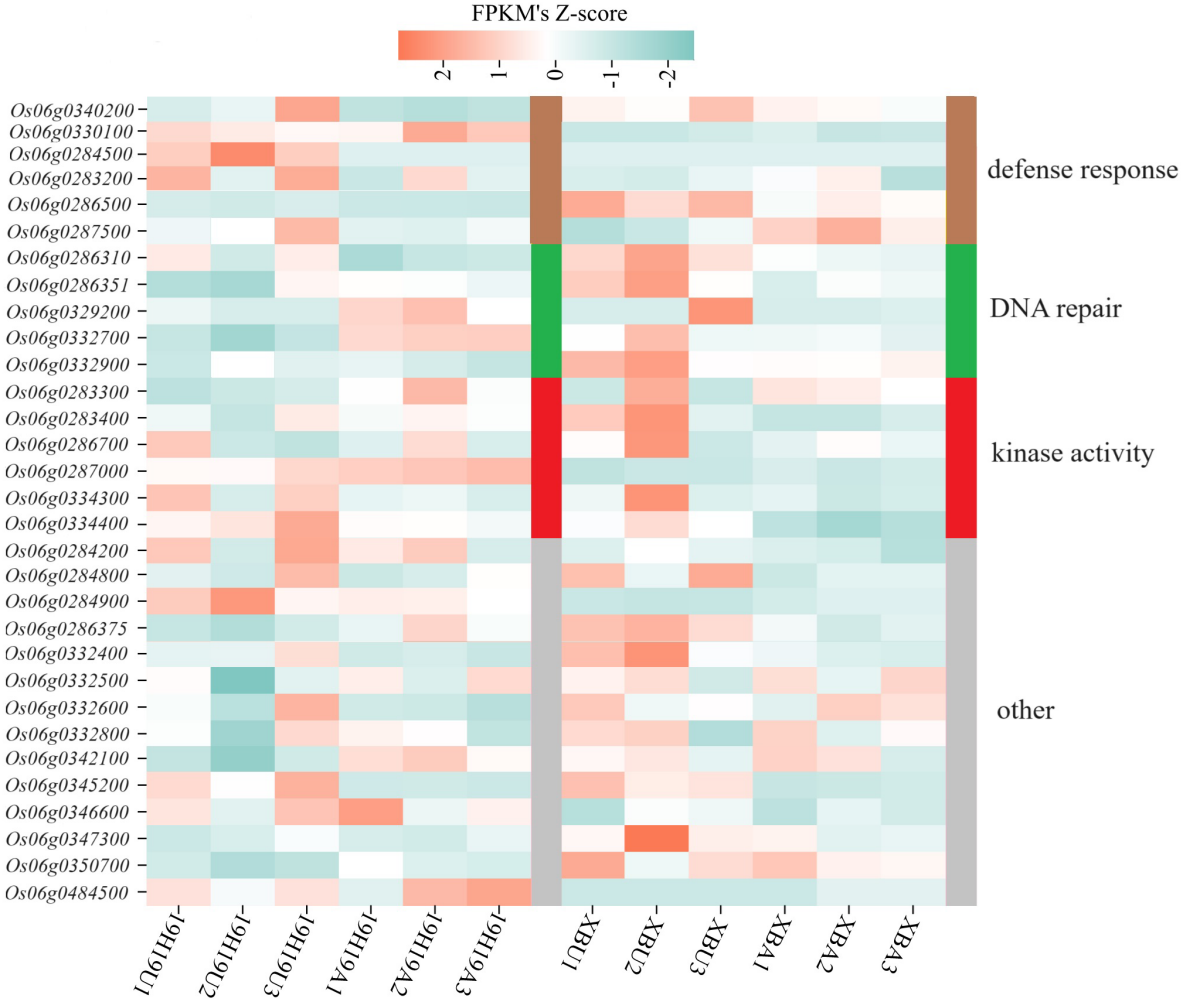
Heatmap of gene expression levels for candidate genes in 19H19 and XB before and after artificial aging. Transcript abundance levels are shown for unaged and aged seeds of 19H19 (19H19U, 19H19A) and XB (XBU, XBA), as determined by RNA-seq.

Based on functional annotation, the 31 genes were classified into 4 categories: 6 related to ‘defense response,’ 5 to ‘DNA repair,’ 6 to ‘kinase activity,’ and 14 to ‘other’ functions (**Figure 4**). Expression levels of ‘DNA repair’ and ‘kinase activity’ related genes showed opposite patterns between XB and 19H19 before and after aging. However, genes related to ‘defense response’ showed inconsistent expression patterns between XB and 19H19: these genes were differentially expressed in 19H19 but not significantly changed in XB before and after aging (**Figure 4**).

### 3.6 Interactions of the genes in the *qSS6.1* interval with others for SS

BSA-seq and QTL mapping collectively identified *qSS6.1*, which had six genes found to interact with the DEGs revealed by RNA-seq: *Os06g0284500*, *Os06g0283400*, *Os06g0287500*, *Os06g0340200*, *Os06g0334300*, and *Os06g0334400*, located in the *qSS6.1* and *qSS6.2* intervals, respectively.

**Figure 5:**
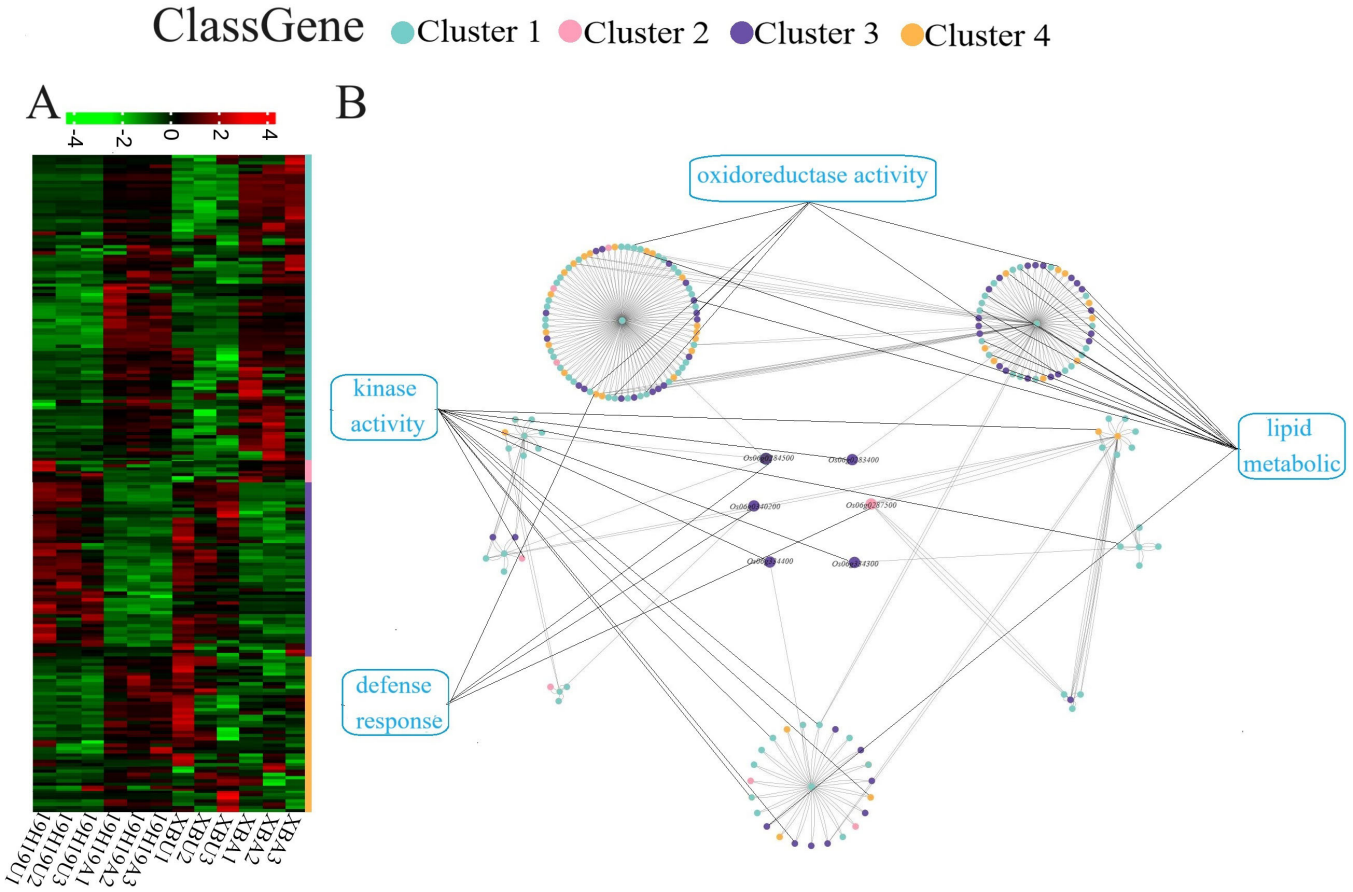
Hierarchical clustering and gene interaction network of candidate genes and differentially expressed genes (DEGs). **(A)** Hierarchical clustering of gene expression profiles in 19H19U, 19H19A, XBU, and XBA based on RNA-seq data from three replicates. **(B)** Interaction network illustrating predicted interactions between candidate genes identified by BSA-seq and DEGs identified by RNA-seq. Candidate genes are shown in the center, and boxes and nodes are colored according to gene function, including categories such as ‘oxidoreductase activity,’ ‘lipid metabolism,’ ‘kinase activity,’ and ‘defense response.

The expression patterns of these interacting genes were mainly classified into four clusters: 1) both 19H19 and XB showed an increasing expression before and after aging; 2) 19H19 had a decreasing expression, whereas XB had an increasing expression after aging; 3) both 19H19 and XB showed a decreasing expression before and after aging; and 4) 19H19 had an increasing expression, whereas XB had a decreasing expression after aging (**Figure 5A**).

Most of the DEGs belonged to Cluster 1 and Cluster 3, exhibiting a trend of consistent expression in both 19H19 and XB before and after aging. The six candidate genes interacting with DEGs were related to ‘oxidoreductase activity’, ‘lipid metabolism’, ‘kinase activity’, and ‘defense response’ (**Figure 5B**; **Supplementary Table S7**). Among them, most were related to ‘lipid metabolism’ and ‘kinase activity’ genes (**Figure 5B**).

### 3.7 Verification of RNA-seq data with RT-qPCR

To verify the reliability of RNA-seq results, the aged seeds from the same batch used for RNA-seq were used for RT-qPCR analysis on the six candidate genes in the *qSS6.1* interval found to interact with the DEGs for SS. The expression differences of these genes were examined before and after aging in 19H19 and XB. The results of RT-qPCR were consistent with those of RNA-seq analysis (**Supplementary Figure S6**), verifying the credibility of the RNA-seq results.

Among them, *Os06g0283400*, *Os06g0284500*, *Os06g0340200*, *Os06g0334400*, and *Os06g0334300* were downregulated (**Supplementary Figure S6**). However, *Os06g0287500* was downregulated in 19H19 but upregulated in XB after aging (**Supplementary Figure S6**). Its differential expression between 19H19 and XB may be caused by differences in promoter sequences (**Supplementary Figure S7**).

*Os06g0287500* had a very low and similar level of expression in both 19H19 and XB before aging, but after aging, 19H19 and XB showed different expression patterns: 19H19 maintained a lower level than before aging, while XB exhibited a much higher expression level than before aging. In contrast, *Os06g0283400* had a very high and similar level of expression in both 19H19 and XB before aging, but after aging, both showed reduced expression levels—although the reduction in 19H19 was less than that in XB (**Supplementary Figure S6**).

### 3.8 Verification of candidate genes

To validate the results of BSA-seq, Sanger sequencing revealed that candidate genes *Os06g0283400* and *Os06g0287500* within the *qSS6.1* interval had non-synonymous mutations or deletions in their exon regions. A non-synonymous mutation in *Os06g0283400* was a change of the nucleotide “A” to “G” in XB (**Supplementary Figure S8A**), while in *Os06g0287500*, it was a deletion of 1-bp “G” in the exon of the gene in XB (**Supplementary Figure S8B**). *Os06g0283400* and *Os06g0287500* are functional genes associated with ‘kinase activity’ and ‘defense response,’ respectively.

Furthermore, the expression levels of genes related to ‘DNA repair’ were opposite in XB and 19H19 seeds before and after aging (**Figure 4**). These findings confirm the credibility of BSA-seq. Further, single marker analysis was performed to investigate the genetic effects of the two genes on SS using their closely linked markers. The identified markers were used to genotype 120 BC_5_F_2_ BILs. PCR amplification was conducted using a thermocycler (ThermoFisher, USA). Accordingly, the LOD score for *Os06g0287500* was 3.70 with a PVE of 19.57 (**Supplementary Table S8**), whereas, *Os06g0283400* showed no significant effect on SS (**Supplementary Table S8**).

**Figure 6:**
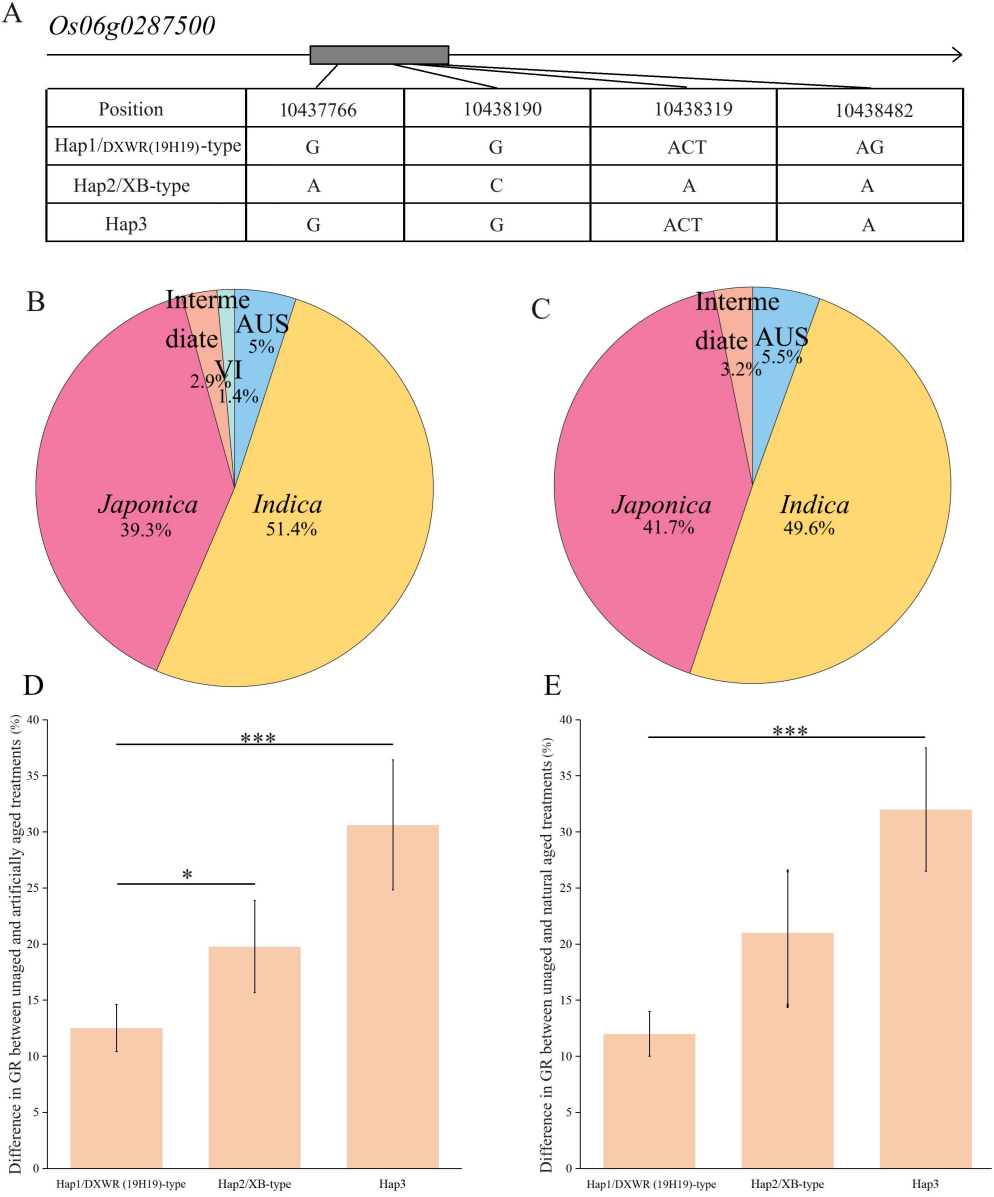
Haplotype analysis of candidate gene *Os06g0287500* in the *qSS6.1* interval. **(A)** Variations in the coding region of *Os06g0287500* across three haplotypes. **(B)** Proportion of rice subspecies types in 141 core accessions. **(C)** Proportion of rice subspecies types in 127 core accessions. **(D)** Differences in germination rate (GR) before and after artificial aging treatment among haplotypes. **(E)** Differences in GR before and after natural aging treatment among haplotypes. Statistical significance was evaluated using Student’s *t*-test *P<0.05, ***P<0.001.

The *Os06g0287500* gene contains only one exon region, and sequencing results indicated two non-synonymous SNPs and two InDels in XB, but not in 19H19 (**Figure 6A**). Screening 141 and 127 core accessions from the RiceVarMap v2.0 database for the four variations in the coding regions of *Os06g0287500* between 19H19 and XB revealed a total of three haplotypes—Hap1, Hap2, and Hap3 (**Figure 6A**). Among the 141 accessions, 51.4% were *indica*, 39.3% were *japonica*, and 5% were Aus; among the 127 accessions, 49.6% were *indica*, 41.7% were *japonica*, and 5.5% were Aus (**Figures 6B, C**). Hap3 occurred most frequently, representing 61.70% and 63.78% of the 141 and 127 accessions, respectively. Hap1 accounted for 31.91% and 30.71%, while Hap2 occurred least, with 6.39% and 5.51% in the 141 and 127 accessions, respectively. 19H19 had Hap1, while XB had Hap2. Accessions in both Hap1 and Hap2 belonged to the *indica* subspecies, whereas Hap3 comprised 7.41% *Aus*, 65.43% *japonica*, and 27.16% *indica* in the 127 accessions. In the 141 accessions, Hap3 comprised 8.05% *Aus*, 63.22% *japonica*, and 28.73% *indica*. A Student’s *t*-test was used to analyze the differences among the three haplotypes, revealing significant variations in SS among them. Hap1 exhibited the strongest SS, demonstrating a preponderant potential for improving SS in rice. The three haplotypes responded differently to the aging treatments. We used the difference in GR before and after aging to measure the response—where the larger the difference, the greater the sensitivity. Under artificial aging conditions, the GR differences were 12.47%, 19.78%, and 31.08% for Hap1, Hap2, and Hap3, respectively. Under natural aging conditions, the GR differences were 12.54%, 20.29%, and 30.72% for Hap1, Hap2, and Hap3, respectively (**Figures 6D, E**). The high similarity between the two aging conditions mutually confirmed that Hap3 showed the greatest response, while Hap1 showed the least. In other words, the least response of Hap1/DXWR(19H19) accessions under either artificial or natural aging conditions demonstrated the strong SS of DXWR. This result confirmed that *Os06g0287500* is a promising candidate gene for SS and that 19H19 is an important germplasm for improving SS in cultivated rice. The *Os06g0283400* gene also contains only one exon region, and sequencing revealed one non-synonymous SNP in XB, but not in 19H19. Screening 141 and 127 core accessions in the RiceVarMap v2.0 database for this variation in the coding region of *Os06g0283400* resulted in two haplotypes—Hap1 and Hap2 —represented by 19H19 and XB, respectively (**Supplementary Table S9**). Hap1 accounted for 24.11% and 23.62% of the 141 and 127 accessions, respectively, while Hap2 occurred most frequently, with 75.89% and 76.38%, respectively. More specifically, Hap1 comprised 37.5% *japonica* and 62.50% *indica* in the 141 accessions. Hap2 comprised 6.73% *Aus*, 42.31% *japonica*, and 50.96% *indica* in the same panel. In the 127 accessions, Hap1 consisted of 40.00% *japonica* and 60.00% *indica*, while Hap2 comprised 7.70% *Aus*, 46.15% *japonica*, and 56.15% *indica*. Under artificial aging conditions, the GR difference was 18.47% for Hap1 and 20.31% for Hap2. Under natural aging, the GR difference was 20.50% for Hap1 and 25.79% for Hap2, respectively (**Supplementary Table S9**). Unfortunately, there was no significant difference in GR between Hap1 and Hap2 under either aging condition.

## 4 Discussion

### 4.1 Importance of DXWR in improving cultivars for SS

Rice seed storability (SS) is essential for grain safety and food security, so the lack of high SS in hybrid rice has been a dilemma and challenge (Chen et al., 2022a). Most modern rice loses seed viability as well as its commercial value very easily during long-term storage (Zhou et al., 2024a). Even worse, the germination rate (GR) of hybrid rice can decrease to 70% or less after one year of storage in southern China, thus resulting in significant losses of various values (Chen et al., 2022a).

In the present study, after artificial aging, 19H19 and XB had 70% and 2% GR, respectively. The high level of GR demonstrates that 19H19 is important germplasm for studying SS in rice, because a GR of 60% after aging is considered a critical value to maintain genetic integrity (Zhou et al., 2023). As a result, 19H19, XB, and their BC_5_F_2_ BIL population were employed to dissect the mechanisms underlying SS by integrating QTL mapping, BSA-seq, and RNA-seq analyses. SS, also determined by the aging index, was calculated as: [GR of unaged seeds-GR of artificially aged seeds)/GR of unaged seeds] x 100%. DXWR has an aging index of 65.9%, tremendously lower than that (100%) of cultivated rice, and 75% of wild rice accessions have strong SS, whereas only 12.8% and 0% of *indica* and *japonica* accessions have strong SS, respectively (Jiang et al., 2010). This shows that DXWR has strong SS and can be a precious germplasm for improving SS in cultivated rice. It further confirms that wild rice species possess stronger SS than cultivated rice (Zhao et al., 2021; Jiang et al., 2010) and that the *indica* subspecies is more storable than *japonica* (Wu et al., 2021). Probably, SS weakens or is even lost during domestication from the wild in rice, especially in the pursuit of high yield through relentless artificial selection. In addition to species, the origin and natural habitat greatly affect the SS of rice germplasm. Rice accessions from tropical and subtropical origins are more storable than those from temperate origins (Wu et al., 2021), which matches the natural adaptation of *indica* and *japonica*. DXWR is the northernmost (116°36′E, 28°14′N) progenitor of cultivated rice and exhibits much stronger SS than modern rice cultivars (Zhao et al., 2021; Zhou et al., 2023; Jiang et al., 2010), making it especially important for cultivar improvement on SS—particularly for *japonica* rice. Notably, the QTL mapping results demonstrate that two of four QTLs in the BC_52_ BILs had favorable alleles from DXWR (*O. rufipogon*) for SS (**Table 1**). Similarly, another study demonstrates that 14 out of 36 QTLs (38.89%) associated with seed vigor under aging treatment come from AA-genome wild rice species *O. longistaminata* (Jin et al., 2018). Wild relatives of cultivated rice harbor beneficial alleles/genes that are absent in cultivated rice, which could be exploited to broaden the narrow gene pool in cultivars. Over the years, much effort has been made to exploit desirable allelic variations from wild rice species for trait improvement, including resistance to biotic and abiotic stresses (Yu et al., 2022). The SS of hybrid rice varieties depends mainly on the male sterile lines for their seed maturation status and storage conditions (Gao et al., 2016; Li et al., 2024). Therefore, developing sterile lines with strong SS is the most promising and effective approach to address the seed storage problem confronting hybrid rice production (Li et al., 2024). Our study for identifying and utilizing favorable DXWR alleles via the maintainer line XB would facilitate genetic improvement of SS in its male sterile line, Xieqingzao A, as well. These results prove that wild relatives of cultivated rice could provide the precious genes for improving SS in *O*. *sativa*.

### 4.2 Identification of three novel QTLs for SS

A genetic map built with 2,059 SNP bin markers identified four QTLs—*qSS6.1*, *qSS7.1, qSS7.2*, and *qSS10*—for SS in our study. Furthermore, BSA-seq identified four QTLs for SS: *qSS4*, *qSS6.1*, *qSS6.2*, and *qSS6.3. qSS6.1* was co-identified by both genomic strategies. Based on the physical positions defined by flanking markers for a QTL associated with germination rate under both natural and artificial aging, only *qSS7.2* co-localizes with the earlier QTL for seed longevity in RC7 (**Table 1**) (Sasaki et al., 2005). *qSST7.1* is associated with germination speed after artificial aging (Liu et al., 2022), while in our study it was linked to germination rate after artificial aging. *qSS4*, located in the 11.76–12.28 Mb region, co-localizes with the previously identified QTL *qSSh4*, which was associated with germination rate under natural aging (Hang et al., 2015). *qSS6.2* and *qSS6.3* both co-localize with the region where *qSS6* was identified for germination rate under artificial aging (Li et al., 2012). To date, only three QTLs for SS have been identified on chromosome 10: *qSST10.1*, *qSST10.2*, and *qSS10* (Dong et al., 2017; Liu et al., 2022). *qSS10*, located in the bin marker interval 103,992,799–108,396,755 in our study, is 1.07 Mb away from *qSST10.2* (Dong et al., 2017). Therefore, *qSS6.1*, *qSS7.1*, and *qSS10* detected in the present study are novel QTLs for SS, expressed by germination rate under artificial aging. The three novel QTLs, particularly those carrying favorable wild rice alleles, can be directly introduced into cultivated rice by crossing the BIL lines. Their application will significantly support the utilization of *O*. *rufipogon* and enrich the genetic basis of cultivars if employed in rice breeding practice.

### 4.3 Importance of gene *Os06g0287500* in improving cultivars for SS

Analysis of DEGs is a proven approach for studying genes associated with a given trait (Lamb et al., 2006) and can effectively reduce the number of candidate genes (Guo et al., 2019). In our study, RNA-seq revealed that 31 out of 53 identified candidate genes exhibited differential expression between XB and 19H19 before and after aging (**Supplementary Table S7**). Furthermore, the expression levels of genes related to ‘DNA repair,’ ‘defense response,’ and ‘kinase activity’ differed between XB and 19H19 before and after aging. This suggests that variation in expression levels of candidate genes in these three functional categories may be closely related to their differences in SS. The gene *Os06g0287500*, located in the *qSS6.1* interval, was one of six genes found to interact with the DEGs. It was downregulated in 19H19 but upregulated in XB after aging (**Supplementary Figure S6**). Functionally, *Os06g0287500* is associated with ‘defense response’ in our study. Haplotype analysis of *Os06g0287500* revealed that rice accessions of the Hap1/DXWR (19H19) type had significantly stronger SS than those of the Hap2/XB type and Hap3 (**Figure 6D**). Interestingly, both Hap1 and Hap2 accessions belonged to *indica*, whereas Hap3 accessions were composed of 63.22% *japonica*. Among the three haplotypes, Hap3 had the weakest SS under both artificial and natural aging conditions. This further confirms that *japonica* rice generally has weaker SS than *indica*, consistent with previous reports (Wu et al., 2021). In the interaction network, DEGs related to ‘kinase activity’ were found to interact with *Os06g0287500* (**Figure 5**). Kinase proteins have been shown to positively regulate seed longevity in *Arabidopsis* (Chen et al., 2022b; Pitorre et al., 2020). Therefore, *Os06g0287500* represents a strong candidate for further gene cloning and molecular improvement of SS in rice. In subsequent studies, we will conduct in-depth investigations into its function and molecular mechanisms to enhance its application in rice breeding.

It is well known that the introgression of favorable alleles from *O*. *rufipogon* may also bring in some unfavorable alleles due to tight linkage in rice. However, the unique QTLs/genes for SS identified from *O*. *rufipogon* in the present study could help develop male sterile lines with improved SS through marker-assisted selection or QTL pyramiding—especially for the gene *Os06g0287500*.

### 4.4 Involvement of hormone-related genes in the regulation of SS in rice

In the present study, the upregulated DEGs unique to 19H19A vs. 19H19U were mainly enriched in ‘auxin biosynthesis’ and ‘gibberellin biosynthesis,’ while the downregulated DEGs were highly enriched in ‘response to abscisic acid (ABA).’ Furthermore, the expression of genes related to ‘auxin biosynthesis,’ ‘gibberellin biosynthesis,’ and their related genes was upregulated in 19H19 after aging, while the genes related to ‘response to ABA’ were downregulated after aging (**Supplementary Figure S5**). However, these plant hormone-related genes did not show differential expression in XB before and after aging treatment.

Plant hormones play a crucial role in regulating SS in rice (Yan et al., 2024), where auxin and gibberellin positively regulate SS, while ABA negatively regulates SS (Wang et al., 2022b; Pellizzaro et al., 2020). Moreover, the indole-3-acetic acid (IAA)-amido synthetase gene *GRETCHEN HAGEN3-2* (*OsGH3-2*) and the ABA catabolism gene *abscisic acid 8’-hydroxylase 2* (*OsABA8ox2*) functionally decrease SS in rice (*Oryza sativa* L.) by modulating the ABA production pathway (Yuan et al., 2021) and the ABA degradation pathway (Zheng et al., 2024a), respectively, both of which increase ABA accumulation. The DEGs for SS identified in our study functionally aligned with previous reports, confirming that the genetic mechanism of SS is regulated by hormone-related genes.

## 5 Conclusion

DXWR exhibits stronger SS than cultivated rice and therefore serves as a precious genetic resource for improving SS in rice cultivars. However, the molecular mechanism underlying SS in DXWR remains unclear, which limits its effective utilization in cultivar improvement. *qSS6.1*, *qSS7.1*, and *qSS10* detected in the present study are novel QTLs for SS expressed by germination rate under artificial aging, based on BSA-seq and genetic linkage analyses. The candidate genes identified were grouped into four functional categories. Among them, the expression level of *Os06g0287500* was downregulated in 19H19 but upregulated in XB after aging due to non-synonymous mutations and deletions in the exon of parental XB. Haplotype analysis of *Os06g0287500* revealed that the SS of Hap1/DXWR (19H19)-type rice accessions was significantly stronger than that of the Hap2/XB type and Hap3. *Os06g0287500* is thus a promising candidate gene involved in SS in rice. These results demonstrate that wild relatives of cultivated rice provide precious genes for improving SS in *O. sativa*.

## Data Availability

The raw RNA-seq data have been deposited in Sequence Read Archive (SRA) (https://www.ncbi.nlm.nih.gov/sra/PRJNA1286829).
