# Supplementary material for: Integrating QTL mapping, BSA-seq and RNA-seq to identify candidate genes regulating seed storability from Dongxiang wild rice
Source: Front Plant Sci. 2025 Aug 13;16:1644153. doi: 10.3389/fpls.2025.1644153 (PMC12380901; doi:10.3389/fpls.2025.1644153)
Supplement: Supplementary file 2 [file DataSheet1.docx]

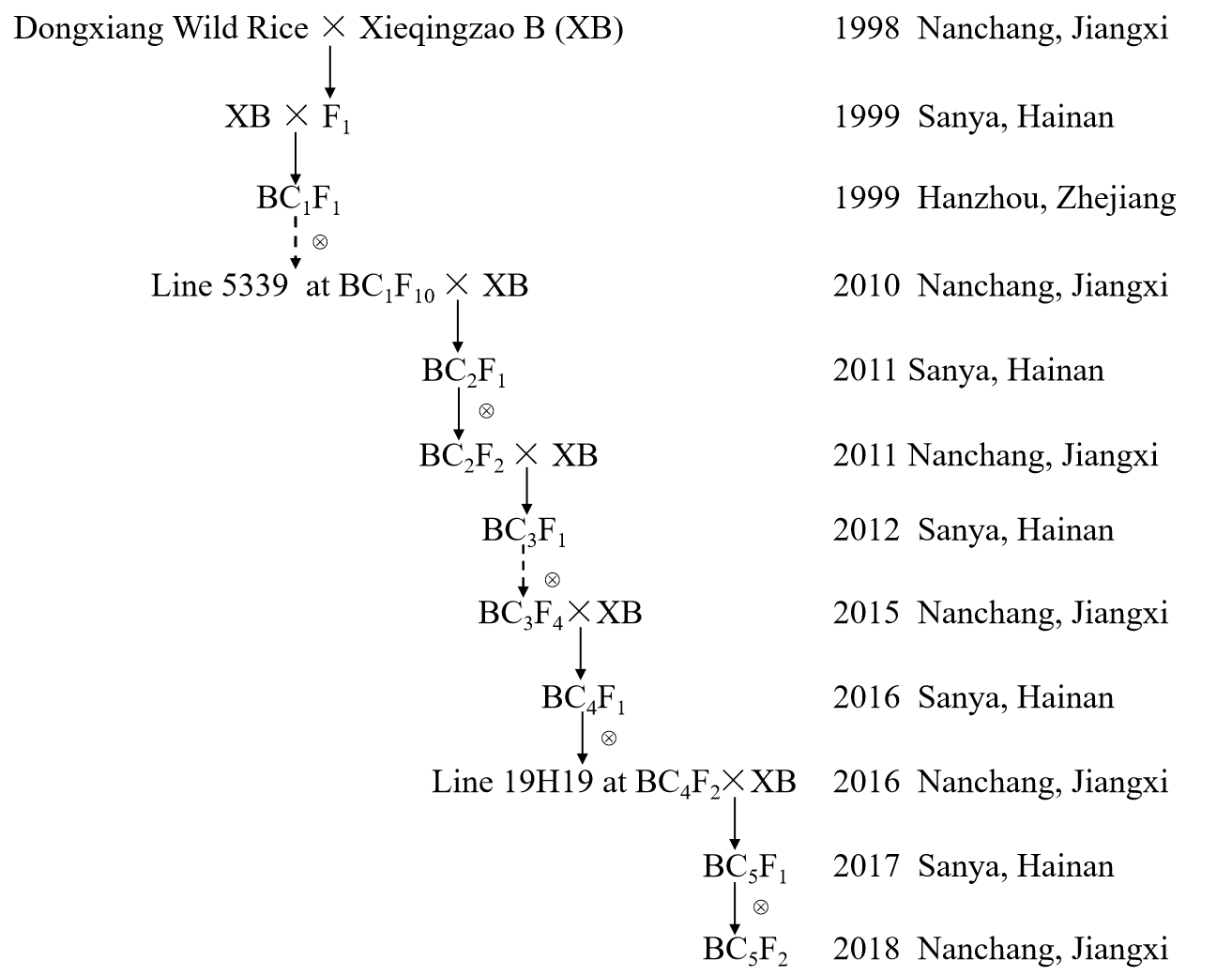


**Fig. S1** Construction diagram of the 19H19 and BC_5_F_2_ populations.


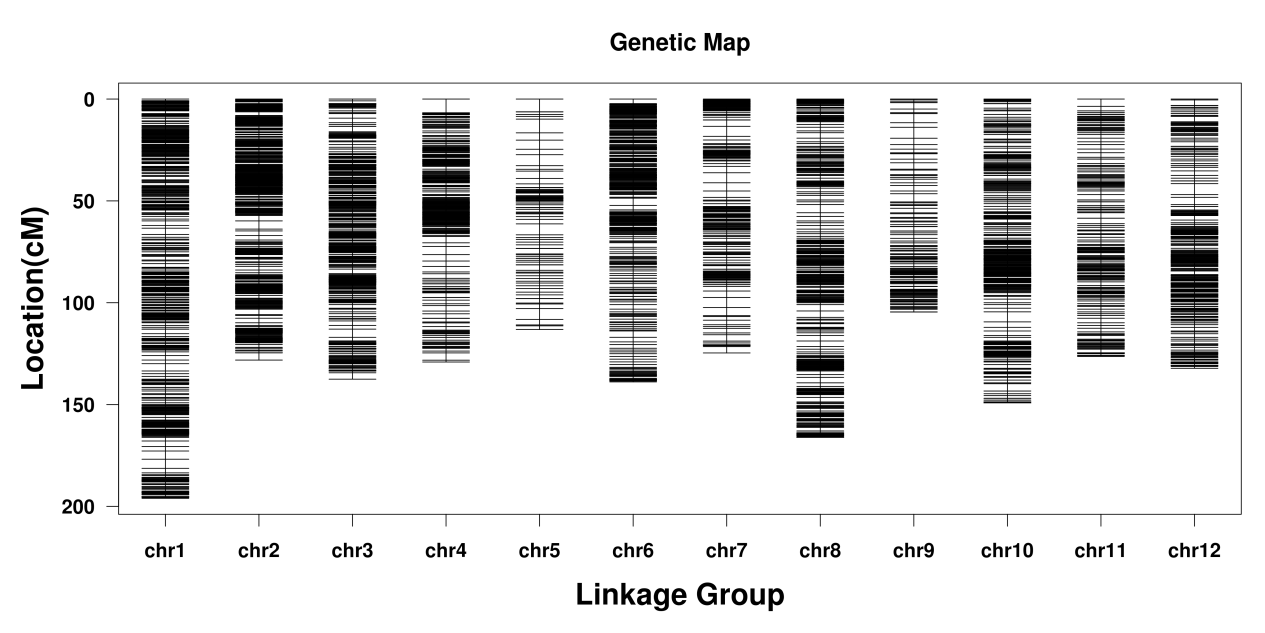


**Fig. S2** The high-density linkage map of rice. A black bar indicates a SNP Bin marker. The x-axis represents the linkage group number and the y-axis indicates the genetic distance (cM) within each linkage group.


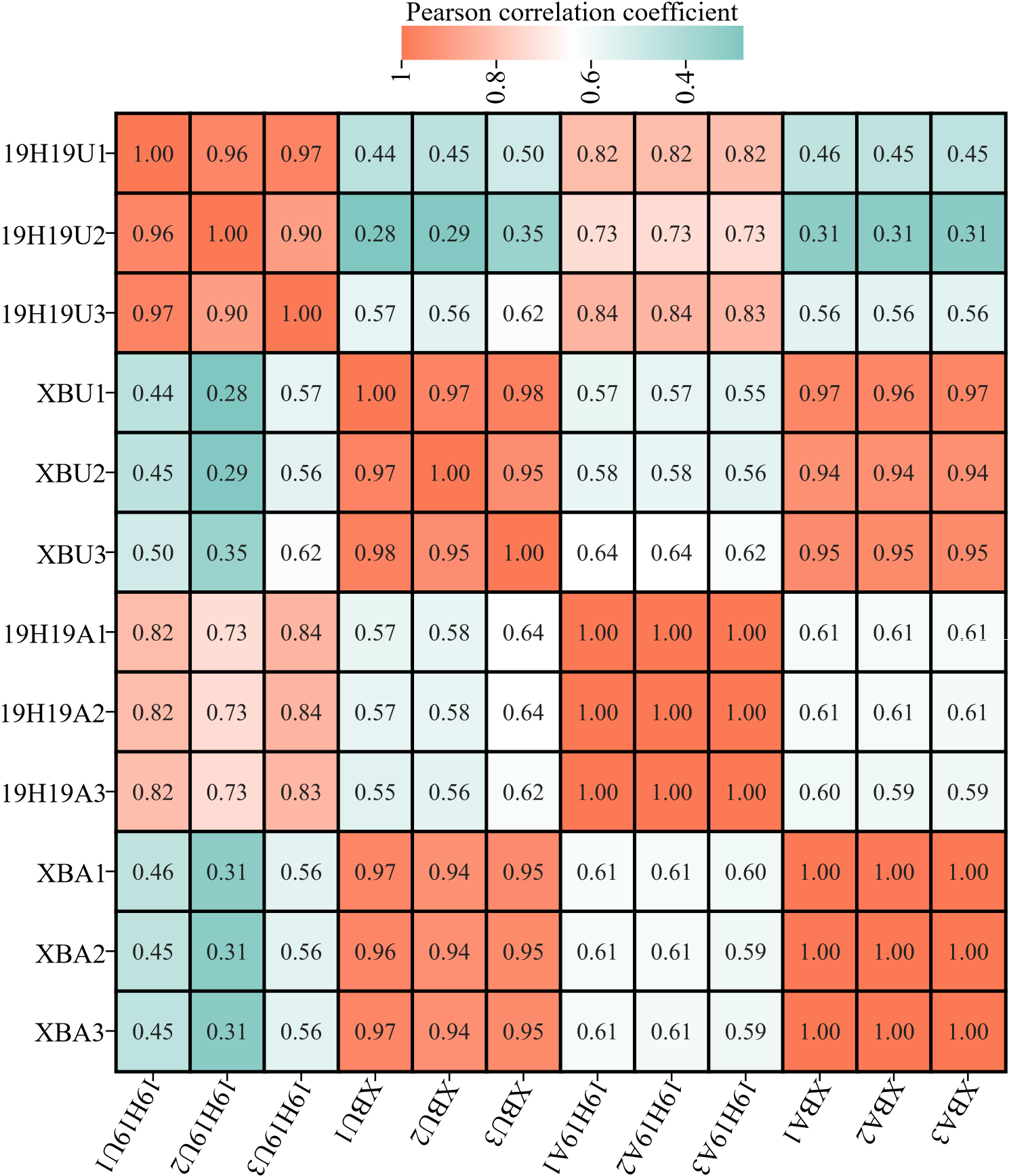


**Fig. S3** Gene-sample correlation analysis.


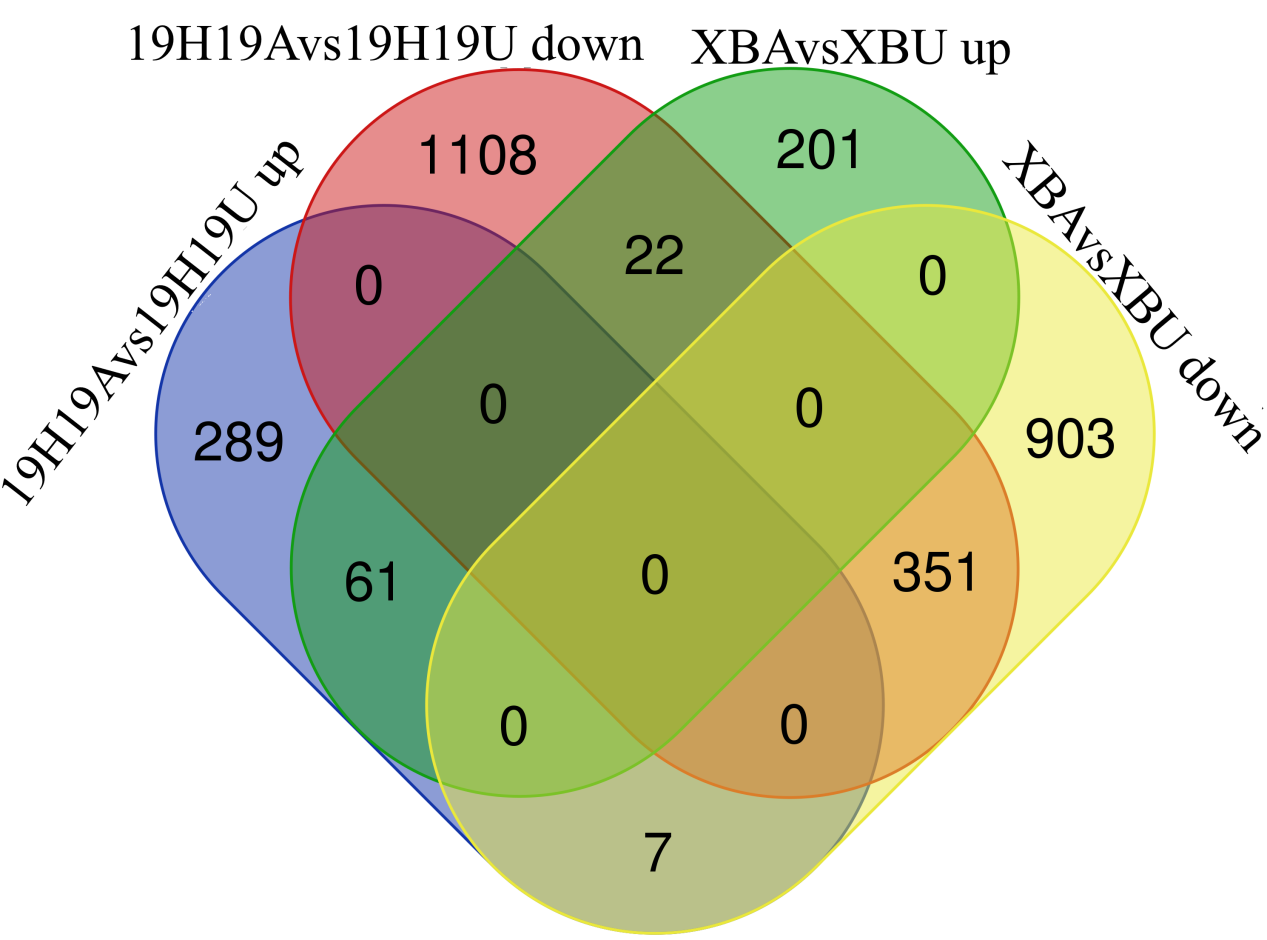


**Fig. S4** Venn diagram showing the numbers of DEGs identified in unaged and artificially aged seeds of 19H19 and XB. Numbers of DEGs identified in the artificially aged vs unaged treatments of 19H19 seeds (19H19A vs. 19H19U) and XB seeds (XBA vs. XBU).


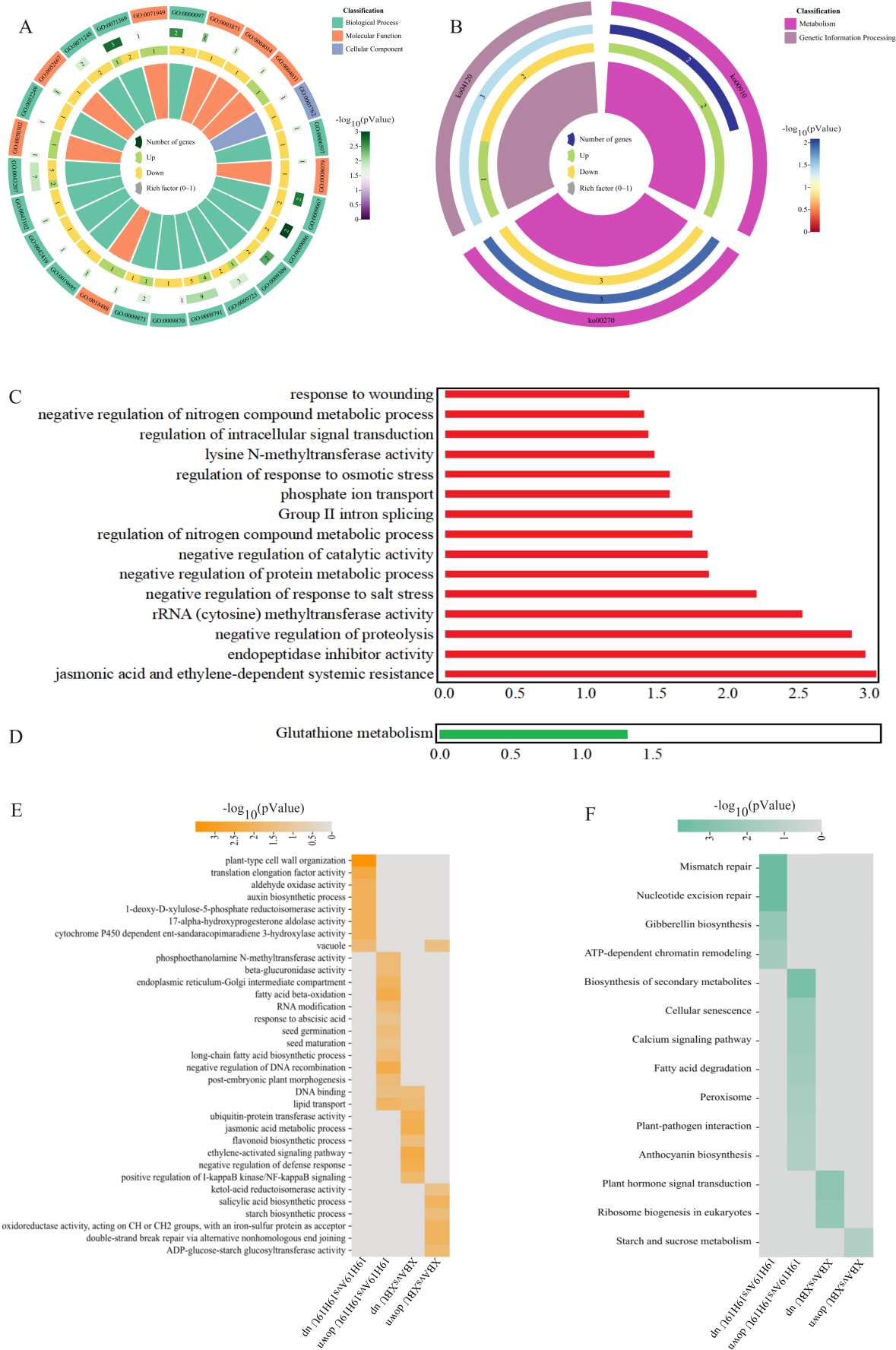


**Fig. S5** GO and KEGG enrichment analyses of DEGs identified in the 19H19Avs19H19U and XBA vs. XBU groups. (A, B) GO (A) and KEGG (B) enrichments of DEGs common to the 19H19A vs. 19H19U and XBA vs. XBU groups. (C, D) GO (C) and KEGG (D) enrichments of DEGs with opposite expression patterns in the 19H19A vs.19H19U and XBA vs. XBU groups. (E, F) GO (E) and KEGG (F) enrichments of DEGs unique to the 19H19LT vs. 19H19RT and XBLT vs. XBRT groups.


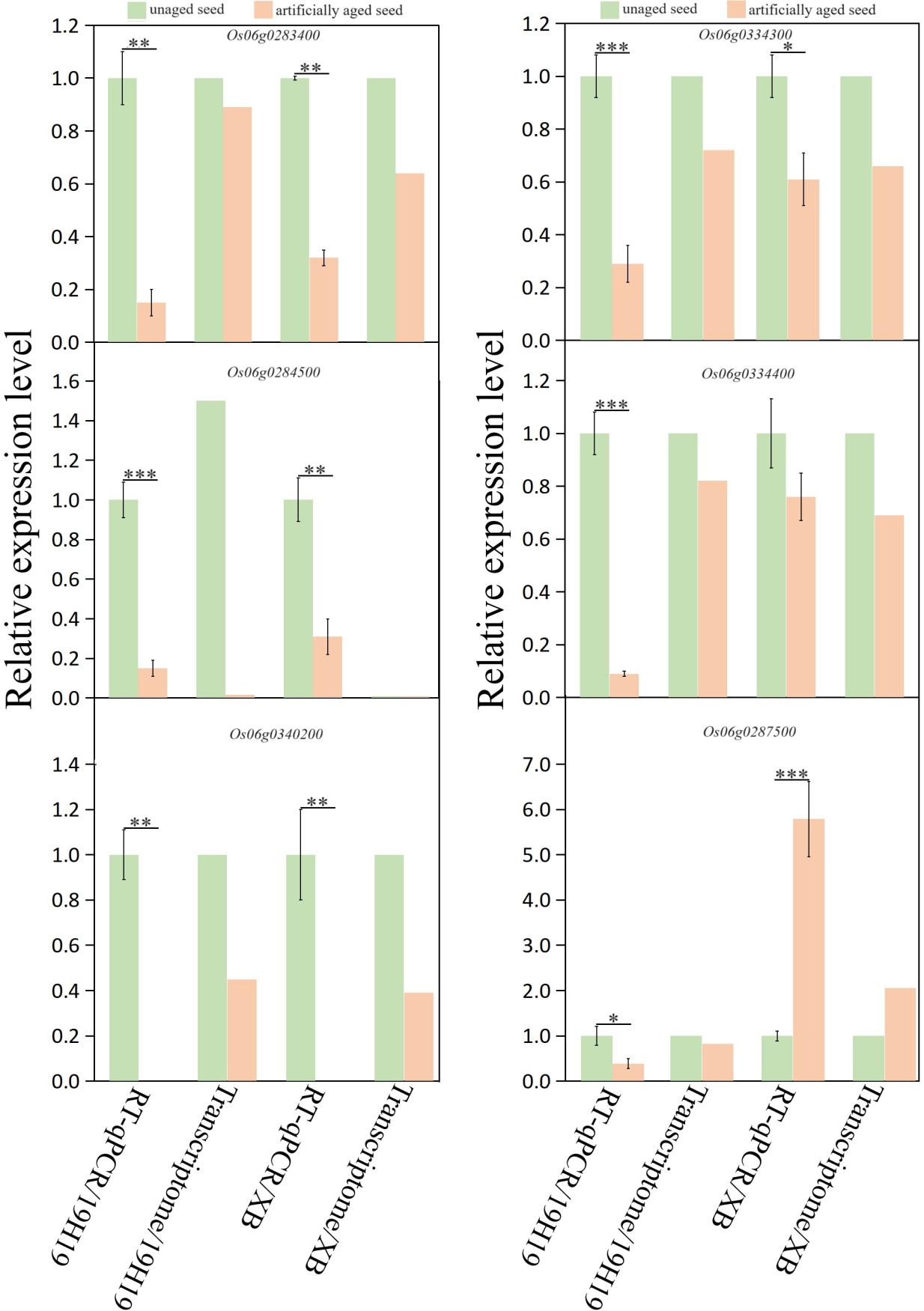


**Fig. S6** Validation of the RNA-seq data of 6 differentially expressed candidate genes of BSA-seq by RT-qPCR. Data represent mean ± SD (n = 3). Significant differences in gene expression levels among the three rice genotypes were determined by Student’s test (**P*< 0.05, ***P*< 0.01, ****P*< 0.001). The expression level of each gene in unaged 19H19 and XB seeds were defined as 1.


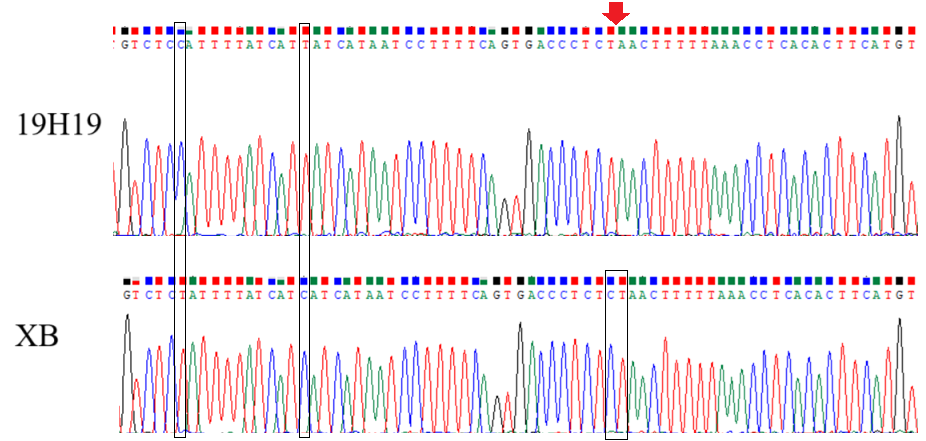


**Fig. S7** Sequencing results of *Os06g0287500* promoter in 19H19 and XB.


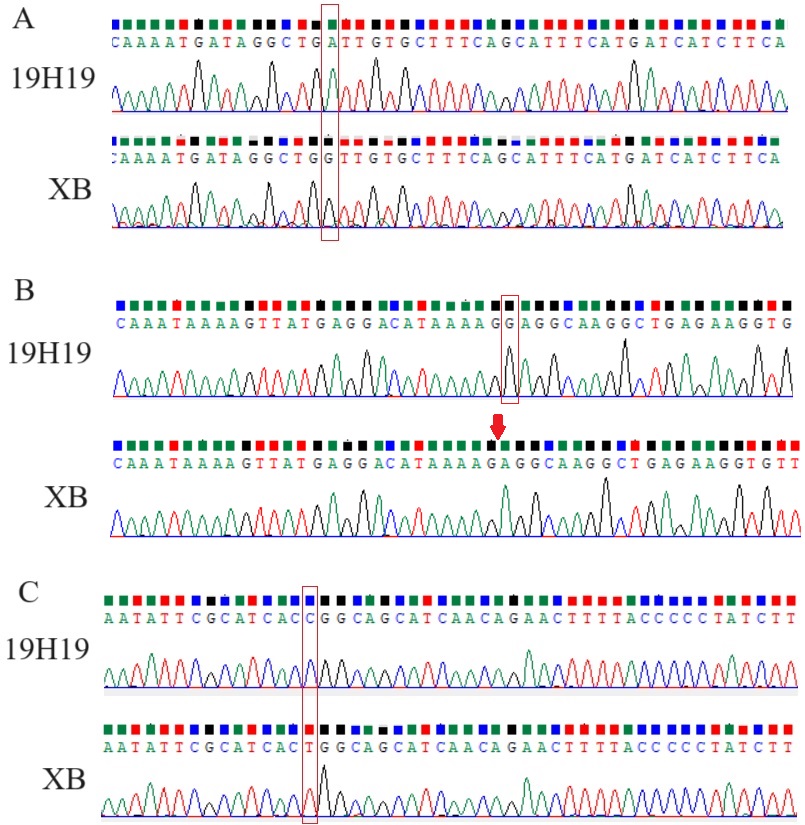


**Fig. S8** The sequencing results of *Os06g0283400* and *Os06g0287500* in 19H19 and XB. (A) *Os06g0283400*; (B) *Os06g0287500*. Red arrows represent the deleted bases, inserted nucleotide is indicated with black boxes and red boxes indicate non synonymous mutations.
